# High presence/absence gene variability in defense-related gene clusters of *Cucumis melo*

**DOI:** 10.1186/1471-2164-14-782

**Published:** 2013-11-12

**Authors:** Víctor M González, Núria Aventín, Emilio Centeno, Pere Puigdomènech

**Affiliations:** 1 Molecular Genetics Department, Center for Research in Agricultural Genomics CRAG (CSIC-IRTA-UAB-UB), Campus UAB, Edifici CRAG, Bellaterra (Cerdanyola del Vallès), 08193 Barcelona, Spain; 2 Bioinformatics Core Unit, Center for Research in Agricultural Genomics CRAG (CSIC-IRTA-UAB-UB), Campus UAB, Edifici CRAG, Bellaterra (Cerdanyola del Vallès), 08193 Barcelona, Spain

## Abstract

**Background:**

Changes in the copy number of DNA sequences are one of the main mechanisms generating genome variability in eukaryotes. These changes are often related to phenotypic effects such as genetic disorders or novel pathogen resistance. The increasing availability of genome sequences through the application of next-generation massive sequencing technologies has allowed the study of genomic polymorphisms at both the interspecific and intraspecific levels, thus helping to understand how species adapt to changing environments through genome variability.

**Results:**

Data on gene presence/absence variation (PAV) in melon was obtained by resequencing a cultivated accession and an old-relative melon variety, and using previously obtained resequencing data from three other melon cultivars, among them DHL92, on which the current draft melon genome sequence is based. A total of 1,697 PAV events were detected, involving 4.4% of the predicted melon gene complement. In all, an average 1.5% of genes were absent from each analyzed cultivar as compared to the DHL92 reference genome. The most populated functional category among the 304 PAV genes of known function was that of stress response proteins (30% of all classified PAVs). Our results suggest that genes from multi-copy families are five times more likely to be affected by PAV than singleton genes. Also, the chance of genes present in the genome in tandem arrays being affected by PAV is double that of isolated genes, with PAV genes tending to be in longer clusters. The highest concentration of PAV events detected in the melon genome was found in a 1.1 Mb region of linkage group V, which also shows the highest density of melon stress-response genes. In particular, this region contains the longest continuous gene-containing PAV sequence so far identified in melon.

**Conclusions:**

The first genome-wide report of PAV variation among several melon cultivars is presented here. Multi-copy and clustered genes, especially those with putative stress-response functions, were found to be particularly affected by PAV polymorphisms. As cucurbits are known to possess a significantly lower number of defense-related genes compared to other plant species, PAV variation may play an important role in generating new pathogen resistances at the subspecies level. In addition, these results show the limitations of single reference genome sequences as the only basis for characterization and cloning of resistance genes.

## Background

Changes in the copy number of DNA sequences have been identified as one of the main mechanisms producing genetic variability in eukaryotes [1,2]. These changes may include the duplication of genomes or chromosomes, duplication of genome segments including genes, and even small duplications both in intergenic and in coding regions [3,4]. Examples of phenotypic effects due to this type of genome variation are increasingly found both in plant species and in animal systems, including humans, where a number of genetic diseases have been linked to copy number variation of specific genes [5-7]. The availability of genome sequences of an increasing number of individuals and species, or specific populations within them, has multiplied the examples of this feature of genomic variability.

Genome sequences are being obtained at an increased rate with the systematic application of massive sequencing techniques. In particular, the genome sequences of different plant species are being published. These include species used in genetic or molecular analysis, such as *Arabidopsis thaliana* and *Brachypodium dystachion*, of interest for their evolutionary relation with other plants, or crop plants of interest in agriculture. In this respect one of the questions that can be addressed at this moment through resequencing of the appropriate varieties is the genomic variability of plant species that occurs at both interspecific and intraspecific levels. These data may help to understand how plant species adapt to changing environments including domestication.

Cucurbits are interesting systems to study changes in genome structure for a number of reasons. They form a family of species that has been the object of genetic analysis for a long time, they occupy a particular place in the phylogenetic classification of plant species, and they are an important crop worldwide and a component of the diet of many European and Asiatic countries since historical times. They have relatively compact genomes (between 300 and 450 Mb) and do not seem to have undergone whole genome duplications besides the ancestral one in the origin of flowering plants [8]. It has also been shown that they have a surprisingly low number of genes coding for proteins with similarity to pathogen resistance-related genes [8-10]. The genome sequences of three important cucurbit crops, cucumber, melon, and watermelon, have already been published [8-10].

The analysis of the high intra-specific genetic variation and morphologic diversity of *Cucumis melo* has led to tentatively classify the subspecific variability of this species into several cultivar groups that can be further broadly grouped into two subspecies, *melo* and *agrestis*[11,12]. The published melon reference genome belongs to the double-haploid line DHL92, derived from a cross between two phylogenetically distant melon cultivars: PI 161375 (Songwhan Charmi), cultivar group *conomon*, subspecies *agrestis*, and the T111 (Piel de Sapo) line, cultivar group *inodorus*, of the subspecies *melo*[8]. Resequencing data from both DHL92 parentals as well as from the reference line itself have already been produced using Illumina technology [8]. Here, results are presented on genome variability of melon after resequencing two additional melon accessions corresponding to a cultivated and an old-relative melon variety. The variation in copy number of different genes has been calculated and it appears there are hotspots of variability in a number of clusters corresponding to genes related to pathogen resistance, indicating species adaptation to changing biotic environments through a genomic strategy.

## Results and discussion

### Genome resequencing of two *Cucumis melo* varieties

**Figure 1:**
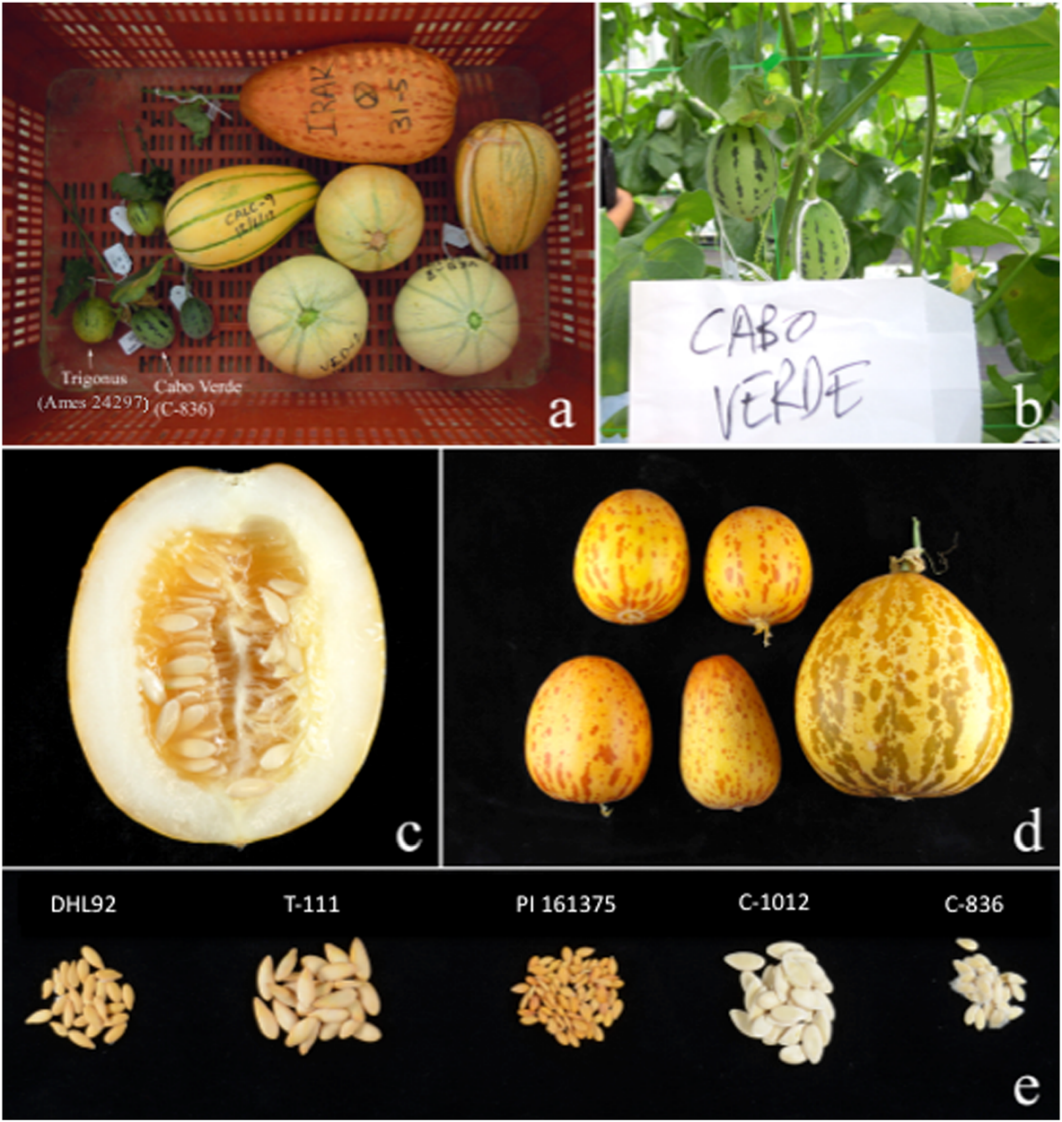
**Fruits from the melon cultivars C-836 and C-1012. (a)** Post-harvest fruits from several melon cultivars, including two *agrestis* varieties (C-836 and C. trigonus, Ames 24297) and one fruit from C-1012, **(b)** 15 day-old C-836 fruits, **(c)** and **(d)** Mature fruits of C-1012, **(e)** Seeds from mature fruits of DHL92, its parental lines, C-836, and C-1012.

To study gene content variations that could be primarily responsible for at least part of the wide phenotypic diversity of melon, two distant melon varieties were chosen for further genome sequencing. One of these cultivars was C-836, an accession from Cabo Verde previously classified as *C. melo* ssp. *agrestis* based on the character of the pubescence type of the female flower hypantium [13,14]. This accession has tiny seeds and very small round fruits (maximum diameter 5–6 cm) with smooth, dark green, black-spotted skin, and non-aromatic flesh (Figure 1a, b). The second variety was C-1012, a cultivar of the subspecies *melo* whose seeds had been collected from a local market in Rawa (Iraq). The fruits of this accession have yellow-orange, smooth skin, very aromatic flesh, and are of medium size (average weight 1 kg, average size 20 × 10 cm) (Figure 1c, d ). The adscription of C-836 and C-1012 to the *agrestis* and *melo* groups, respectively, was confirmed building a phylogenetic tree to position those cultivars relative to five other melon varieties (Additional file 1).

Genomic DNA from young leaves of both cultivars was used to produce two paired-end libraries of average insert size *ca*. 500 bp, subsequently sequenced using the Illumina Genome Analyser IIx technology. Around 30 million pairs of cleaned, high-quality reads of maximum length 152 bp were obtained for each cultivar (Table 1). Similar amounts of Illumina reads previously obtained from DHL92 and its two parental lines [8] were also included in the analysis (Table 1). In all, between 6.1 Gb (C-836) and 7.5 Gb (C-1012) of total sequence length were produced, representing ×13-×16 genomic coverage, assuming a melon genome size of 454 Mb [15]. These paired-end data sets were then aligned to the melon reference genome to produce *bam* files as described in the Methods section.

**Table 1:** Illumina sequencing and pair-end mapping metrics

**Cultivar**	**Reads**^**a**^**(pairs)**	**High-quality reads (pairs)**	**Total length (Mb)**	**N75**^**b**^
**C-836**	28,038,962	23,341,896	6,099.4	128
**C-1012**	33,207,205	27,060,551	7,041.7	126
**DHL92**	35,538,240	28,857,265	7,314.3	122
**PI 161375**	35,233,293	28,809,870	7,424.0	124
**T111**	35,857,911	29,305,082	7,495.4	124

^a^Raw, unfiltered data.

^b^75% of the total length is contained in all reads ≥ N75.

### Detection and functional characterization of PAV genes

In order to detect genes putatively absent from a genomic region in one cultivar, compared to the corresponding region in the reference genome (hereupon referred to as PAV genes), the genomic coordinates of all 27,427 melon annotated genes were used to search the alignment files for regions of very low coverage in at least one of the four melon cultivars analyzed (see Methods section for a detailed account of the screening process). The mapping patterns at the candidate loci were visually inspected to produce a final list of 1,215 putative PAV genes (4.4% of the predicted melon gene complement). This list can be found in Additional file 2: Table S1, together with the information on gene absence distribution for each melon cultivar and gene functional annotations.

Compared to the reference genome, a total of 854 genes (70.3% of all PAV genes) were absent in only one cultivar, while 263 genes (21.7%) were absent in two cultivars, 84 (6.9%) in three cultivars, and only 14 genes were missing in more than three cultivars. In all, 1,697 PAV events were detected, distributed in all cultivars analyzed, including 30 in DHL92, which probably represents mapping artifacts as no PAV would be expected when mapping DHL92 reads against the DHL92 reference genome itself. Only two genes were absent from DHL92 but present in both parental genomes, which clearly indicates false positives for the detection of PAVs.

In addition, not all melon varieties had the same content of PAV genes. In cultivar C-836 there were 788 PAV events (46.4% of all PAV events), while C-1012, T111 and PI 161375 had, respectively, 334 (19.7), 250 (14.7%), and 295 (17.4%) events. However, it must be taken into account that both T111 and PI 161375 are the parental lines of DHL92 and so it is expected that the number of PAV events would be significantly lower than those found when comparing two not so closely related melon cultivars, as each one contributes to around 50% of the reference genome. In all, the average number of absent genes per cultivar was 416 (not including the 30 DHL92 events) and, therefore, *ca*. 1.5% (ranging from 0.9 to 2.9%) of all genes were absent, on average, in the analyzed cultivars as compared to DHL92. These figures are comparable with values published for *Arabidopsis* and rice [16,17].

The functional annotation of the candidate PAV genes was obtained from the MELONOMICS webpage [18]. Also, a Blast2GO analysis was performed to gather further information on genes with unknown function. Additionally, functions were tentatively assigned to ten of these genes using phylogeny-based information from the melon phylome [19], as for the general annotation of the genome [8]. Using this combined approach, and excluding all genes with only generic information available, such as 'ankyrin repeat-containing', 'coiled-coil', 'oxidoreductase activity' and 'catalytic activity', functional information was finally assigned to 387 PAV genes (31.8% of the total) (Additional file 2: Table S1). However, upon analyzing the functional annotations, it was found that 83 of these genes were annotated as encoding transposon-related sequences. The melon reference genome has been annotated after masking mobile elements [8]. The inspection of these 83 sequences showed that 25 were true transposons that somehow had not been masked during the sequencing assembly; the rest did show homology with proteins in our transposon databases but with e-values lower than 1e-10. These sequences probably represent degenerate forms of old mobile elements, transposon fragments that remain in the genome after an excision event, or they are genes that have some degree of similarity to transposon sequences.

The 304 putative PAV genes with functional annotations other than transposon-related sequences were classified according to their ontology (Additional file 3: Table S2). More than 80% of the annotated genes could be grouped into nine categories: amino-acid, carbohydrate and lipid metabolism, biotic or abiotic stress response, terpenoid biosynthesis, transcription/translation processes, transport, and two groups containing generic P450 and protein/DNA- binding proteins that could not be further classified due to lack of more specific information.

Interestingly, the most populated functional category was that of stress response proteins (92 genes, 30% of all classified PAVs), with a group of 25 TIR-NBS-LRR/NB-ARC disease-resistance proteins by far the largest subclass of PAV genes having an associated function. It is known that structural genome variants in plants are prone to affect stress response genes, particularly those for disease resistance [16,20,21]. These genes have been found to be underrepresented in the genomes of cucurbits when compared to those of other plant species.

Previous studies have shown that, although whole-genome duplication events seem to be absent from the lineages leading to *C. melo*, several gene families have expanded specifically in the *Cucumis* genus compared with other cucurbits, or in *C. melo* when compared with *Cucumis sativus L.* (cucumber) [8]. The list of expanded families includes defense response, lipid metabolism, cell wall structure, translation and transport gene families which, as we report above, are also enriched in PAV genes. Crossing the lists of PAV and expanded families to search for coincidences, a total of 10.8% of genes in families that have expanded specifically in the *Cucumis* genus and 16.1% in *C. melo* were found to be PAV. These values are 2.4-3.4 times higher than the abundance of PAV genes, 4.4%, in the melon gene complement.

### Presence of PAV genes in multi-copy gene families and gene clusters

It has been previously suggested that with genes belonging to multi-copy gene families, and especially those arranged in clusters, there is a greater chance of presence/absence polymorphism, probably due to unequal crossover between homologous genes. This seems to be particularly the case for disease resistance genes: there is evidence that clusters of these genes may well be hotspots for the generation of PAV [16,21-23]. In melon, previous studies have shown that resistance genes are frequently found in clusters in the genome; most notably, half of the 81 predicted melon NBS-LRR genes groups within nine clusters [8,24-27]. A region spanning 760 kb of linkage group V [MELO3C004258-MELO3C004325] and containing the *Vat* gene *locus* is of particular interest. It contains 28 NBS-LRR resistance genes, the highest concentration of R genes found in the melon genome. A microsynteny comparison of part of this region and its homolog in the cucumber genome has already shown the existence of PAV polymorphisms affecting several R genes [27].

**Figure 2:**
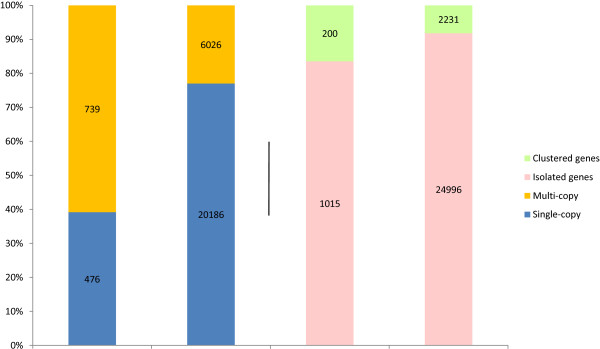
**Distribution of PAV genes in multi-copy gene families and gene clusters.** Number and proportion of PAV and non-PAV genes that can be classified as single-copy/multi-copy and clustered/isolated in the compared melon sequences.

The presence of PAV genes in multi-copy gene families and gene clusters was further investigated to see whether presence/absence variants were associated to these families with higher frequency than randomly expected. To this end, the melon gene complement was first grouped in families of homologous genes as explained in the Methods section. As a result, 75.3% of all genes were classified as belonging to single-member families or singletons, while 6,765 genes were members of 1,573 multi-copy gene families. By comparing the PAV and multi-copy gene lists, it was found that 739 PAV genes (60.8% of all PAV genes) can be classified as multi-copy while 6,026 non-PAV genes (23% of all non-PAV genes) belong to multi-copy families (Figure 2). Therefore, there was a significantly higher proportion (χ^2^ = 892, df =1; p-value < 0.0001) of multi-copy genes in the PAV subset of the melon gene complement. As only 2.3% of singleton genes were classified as PAV compared to 10.9% PAVs in the multi-copy fraction, genes present in the genome in more than one copy are five times more likely to be affected by PAV than singleton genes.

Most of the multi-copy gene families contain only two (1,013 families, 64%) or three (250 families, 16%) genes. These small families represent 41% of all multi-copy genes but only 29% of all multi-copy PAV genes. On the other hand, 97 families of 7–20 gene members contain 15% of all multi-copy genes but 22% of all multi-copy PAV genes. Therefore, PAV genes appear to have a slightly higher tendency to be present in more populated gene families in the melon genome.

To see whether the physical location of analogous genes could also be related to the occurrence of presence/absence polymorphisms, the presence of PAV genes in clusters of genes with a significant degree of similarity was investigated. By classifying multi-copy genes in clusters (as described in the Methods section), a total of 2,431 genes, of which 200 were PAVs, grouped in 987 clusters (Figure 2). Therefore, the proportion of PAV genes clustered in the melon genome (16.5%) is significantly higher than the 8.5% of non-PAV clustered genes (χ^2^ = 92, df =1; p-value < 0.0001). Also, as only 4% of non-clustered genes are PAV compared to the 8% of clustered genes, it seems clear that the chance of genes present in the genome in tandem arrays being affected by PAV is double that of isolated genes. Of all 987 clusters, 77% are just tandems of two genes, and the longest cluster found had 20 genes. Significantly, 14% of all clustered genes, but 28% of all PAVs in tandem, were found in the 47 clusters of five or more genes, indicating that PAVs tend to be present in longer clusters.

### Validation of putative PAV genes

The prediction of PAV genes has been based solely on the analysis of the alignment files, with no specific experimental support. Since many PAV genes belong to medium-large multigene families, there is more than a negligible possibility of mapping artifacts mistakenly taken for putative PAVs. Therefore, it was decided to determine which fraction of putative PAV genes was truly absent from the analyzed cultivars. The validation of PAVs was mostly carried out in a 1.1 Mb region that spans the 760 kb region described above, due to its high level of PAV polymorphisms and multi-copy gene content (see next section for a more detailed description of this region).

Since all reads were mapped against the DHL92 genome, it was important to confirm that no chimeras had been produced during the assembly of this region on the reference genome. The sequence of a 250 kb fragment with the MELO3C004258-4290 genes, previously obtained from three overlapping BACs, was used to validate the corresponding sequence in the assembled reference genome [8,24,26,27]. To validate the MELO3C004290-4331 gene fragment, a tiling path of 10 BAC clones spanning 713 kb and comprising the melon genes MELO3C004287-MELO3C004347 was obtained as described in the Methods section (Additional file 4: Figure S1). The BAC lengths were estimated by averaging 3–7 pulsed-field electrophoretic runs for each clone (Additional file 5: Figure S2).

**Figure 3:**
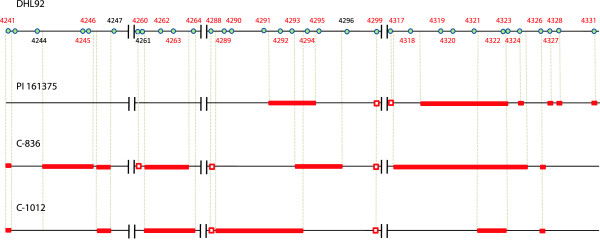
**PAV polymorphisms in the stress-response and PAV gene-rich 1.1 Mb genome fragment.** Red bars represent fragments absent in melon varieties compared to the DHL92 reference sequence. Empty red squares represent partially absent genes. As T111 ('Piel de sapo') is known to be the parental donor of the chromosome fragment containing this region, no PAVs were found for this variety and, therefore, only three cultivars (C-836, C-1012, and PI 161375) and the reference line are shown.

The estimated lengths of eight clones differed by 10 kb on average (7–16 kb) when compared with the expected values obtained by mapping the BAC-end sequences to the reference genome assembly. This result is acceptable bearing in mind both the expected error in the BAC length estimations based on pulsed-field electrophoresis and, more importantly, the abundance of stretches of Ns on the reference genome. Their sizes had been adjusted on the basis of pair-end mapping information during the assembly process and, therefore, can be expected to differ to some extent from the actual lengths of the sequences they represent. In particular, two clones spanning the MELO3C04318-4324 region differed from their expected lengths by only 7 kb, which indicates that the 150 kb and 90 kb fragments absent in that region in the two *agrestis* cultivars (see Figure 3 and Additional file 4: Figure S1) are most probably present in the reference DHL92 sequence.

Two overlapping clones spanning the MELO3C04287-4294 genes were found to be 50–60 kb larger than their expected sizes. As both clones share 36 kb of sequence, this suggests that about 50 kb are missing from the reference genome sequence somewhere in the shared region, which has only one annotated gene (MELO3C04291). In all, the existence of a contig of BACs whose sequence ends map adequately to the reference genome validates the overall sequence structure of, at least, the MELO3C04295-4331 region from DHL92, although minor artifacts cannot be discarded.

In a complementary approach, individual putative PAV regions were confirmed by PCR using primers flanking the candidate regions and genomic DNA from the studied varieties, and BAC or genomic DNA from the reference genome. A total of 40 genes were analyzed, 23 belonging to the 1.1 Mb region of linkage group V. The results confirmed 34 (85%) of the putative PAV genes while six genes that had been considered partially absent were found in the respective cultivars (Table 2).

**Table 2:** PCR validation of PAV polymorphisms

**PAV gene**	**Coordinates (v3.5)**^**a**^	**Partially or fully absent (p/f)**	**Varieties**^**b**^	**PAV gaps**^**c**^		
				**Size (bp)**	**Coordinates (v3.5)**^**a**^	**Comments**^**d**^
**4241**	5,250,561-5,252,741	f	**IQ**	6,225	5,246,795-5,253,019	C
			**CV**			
**4260**	5,508,406-5,509,772	p	**CV**	498	5,508,275-5,508,772	C, SI (37 nt), E
**4262**	5,533,446-5,539,663	p	**IQ**	-	-	False PAV
			**CV**			
**4278**	5,683,128-5,692,676	p	**CV**	1,308	5,684,308-5,686,215	C, I (partial)
**4288**	5,779,041-5,786,300	p	**IQ**	-	-	False PAV
			**CV**			
**4289**	5,801,121-5,806,330	f	**IQ**	86,393	5,790,312-5,876,704	C, SI (3 kb)
		p	**CV**	739	5,802,779-5,803,517	C, I
**4290**	5,810,121-5,812,410	f	**IQ**	86,393	5,790,312-5,876,704	C, SI (3 kb)
**4291**	5,840,867-5,843,162	f	**IQ**	86,393	5,790,312-5,876,704	C, SI (3 kb)
		p	**PI**	57,737	5,843,353-5,901,089	SI (744 nt)
**4292**	5,855,171-5,860,831	f	**IQ**	86,393	5,790,312-5,876,704	C, SI (3 kb)
		p	**CV**	532	5,857,419-5,857,950	C, E (partial), I (partial)
		f	**PI**	57,737	5,843,353-5,901,089	C, SI (744 nt)
**4293**	5,868,071-5,868,244	f	**IQ**	86,393	5,790,312-5,876,704	C, SI (3 kb)
		f	**PI**	57,737	5,843,353-5,901,089	C, SI (744 nt)
**4294**	5,884,952-5,889,556	f	**CV**	40,204	5,878,047-5,918,250	C, SI (3 kb)
		f	**PI**	57,737	5,843,353-5,901,089	C, SI (744 nt)
**4295**	5,893,422-5,893,773	f	**CV**	40,204	5,878,047-5,918,250	C, SI (3 kb)
		f	**PI**	57,737	5,843,353-5,901,089	C, SI (744 nt)
**4299**	5,951,143-5,955,050	p	**IQ**	525	5,953,236-5,953,760	I
		p	**CV**	525	5,953,236-5,953,760	I
		p	**PI**	524	5,953,237-5,953,760	I
**4317**	6,085,745-6,087,399	p	**PI**	-	-	False PAV
**4318**	6,111,948-6,113,580	f	**CV**	149,716	6,088,767-6,238,482	C
**4319**	6,144,630-6,145,072	f	**CV**	149,716	6,088,767-6,238,482	C
		f	**PI**	87,210	6,130,422-6,217,631	C
**4320**	6,158,620-6,160,417	f	**CV**	149,716	6,088,767-6,238,482	CC
		f	**PI**	87,210	6,130,422-6,217,631	C
**4321**	6,180,382-6,182,596	f	**CV**	149,716	6,088,767-6,238,482	C
		f	**PI**	87,210	6,130,422-6,217,631	C
**4322**	6,212,481-6,214,658	f	**CV**	149,716	6,088,767-6,238,482	C
		f	**PI**	87,210	6,130,422-6,217,631	C
		f	**IQ**	-	-	Not yet confirmed
**4323**	6,219,959-6,220,884	f	**CV**	149,716	6,088,767-6,238,482	C
**4324**	6,232,683-6,236,414	p	**IQ**	1,637	6,234,788-6,236,424	C, E (partial)
		f	**CV**	149,716	6,088,767-6,238,482	C
		f	**PI**	5,714	6,232-627-6,238,340	C
**4326**	6,251,955-6,252,158	f	**IQ**	3005	6,250,865-6,253,869	C, SI (5.8 kb)
		f	**CV**	3588	6,250,865-6,254,452	C, SI (6 kb)
**4331**	6,306,224-6,307,985	f	**PI**	6,370	6,306,115-6,312,484	C
**5453**	5,239,146-5,239,370	p	**IQ**	0	5,239,342-5,239,343	C, SI (225 bp)
		p	**CV**	0	5,239,290-5,239,343	
**14632**	4,629,508-4,646,365	p	**CV**	7,278	4,628,501-4,635,778	C, E, I, SI (39 bp)
**14633**	4,655,610-4,656,362	f	**IQ**	33,861	4,646,955-4,680,815	C, SI (7.5 kb)
		f	**PI**	33,861	4,646,955-4,680,815	C, SI (7.5 kb)
**14634**	4,658,485-4,659,820	f	**IQ**	33,861	4,646,955-4,680,815	C, SI (7.5 kb)
		f	**PI**	33,861	4,646,955-4,680,815	C, SI (7,5 kb)
**14635**	4,676,224-4,677,197	f	**IQ**	33,861	4,646,955-4,680,815	C, SI (7.5 kb)
		f	**PI**	33,861	4,646,955-4,680,815	C, SI (7,5 kb)
**15015**	1,061,280-1,061,542	f	**CV**	28,011	1,038,039-1,066,049	C, SI (2,6 kb, transposon)
**22141**	1,707,299-1,710,539	p	**IQ**	-	-	False PAV
		p	**PI**			
**22145**	1,728,890-1,744,072	p	**IQ**	9,736	1,733,997-1,743,732	C, E, I, SI (6.5 kb)
		p	**CV**	9,736	1,733,997-1,743,732	C, E, I, SI (6.5 kb)
		p	**PI**	9,736	1,733,997- 1,743,732	C, E, I, SI (6.5 kb)
**22153**	1,815,300-1,819,596	p	**PI**	-	-	False PAV
**22154**	1,823,379-1,831,379	p	**CV**	-	-	False PAV
		p	**PI**			
**23276**	756,768-760,353	f	**IQ**	19,558	755,353-774,910	C, SI (445 bp)
		f	**PS**	19,558	755,353-774,910	C, SI (310 bp)
**23277**	765,000-765,855	f	**IQ**	19,558	755,353-774,910	C, SI (445 bp)
		f	**PS**	19,558	755,353-774,910	C, SI (310 bp)
**23278**	766,024-767,842	f	**IQ**	19,558	755,353-774,910	C, SI (445 bp)
		f	**PS**	19,558	755,353-774,910	C, SI (310 bp)
**23577**	1,518,347-1,521,271	p	**CV**	2,191	1,519,298-1,521,488	C, E, I
**24733**	294,304-297,983	p	**CV**	193	295,122-295,314	C,I,SI (234 bp, transposon)
**24737**	344,775-345,020	f	**CV**	4,959	344,525-349,483	C
**24743**	468,195-469,071	p	**CV**	726	468,516-469,241	C, E, I

^a^Coordinates on the scaffold/contig that contains the gene.

^b^CV: C-836, IQ: C-1012, PI: PI 161375, PS: T111.

^c^DHL92 sequence fragment that contains the gene and is absent relative to the reference sequence.

^d^C: PAV confirmed; E: Exon deletion; I: Intron deletion; SI: Sequence insertion relative to the reference.

Sequencing of the amplified PCR bands showed that the absence of a genomic fragment was often accompanied by the presence of sequences that were absent from the reference genome, from just a few to several thousand nucleotides. In two instances these new sequences were found to show homology with transposons, indicating the role that mobile elements may play in the generation of PAV polymorphisms [28].

### Analysis of PAVs in a 1 Mb region of linkage group V

**Figure 4:**
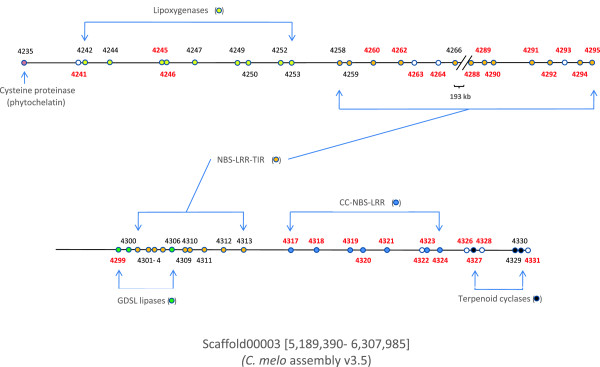
**Distribution of stress-response genes and PAV polymorphisms in a 1.1 Mb fragment of linkage group V.** Genes are represented by circles. Only putative stress-response genes and clusters of genes with at least one PAV are shown. Gene ID numbers in red denote PAV genes. A 193 kb internal fragment without PAV or stress-response genes has been omitted. Figure drawn to scale.

**Figure 5:**
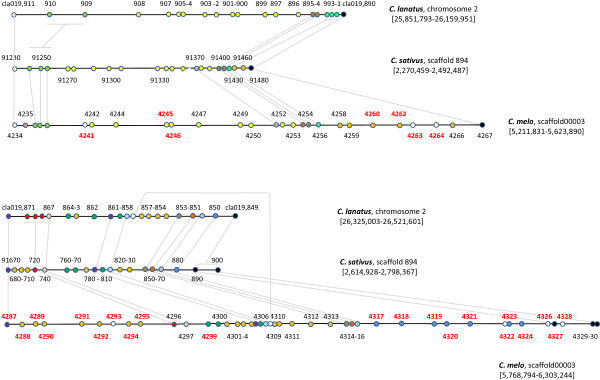
**Overview of microsynteny between the stress-response and PAV gene-rich 1.1 Mb melon genome fragment and its homologous sequence in cucumber (*Cucumis sativus*) and watermelon (*Citrullus lanatus*).** Genes are represented by circles. Genes with the same biological function are shown in the same color. Colors represent either multi-copy genes (intra-specific) or syntenic genes (inter-specific): yellow circles, lipoxygenase genes; orange circles, TIR-NBS-LRR genes; dark green circles, GDSL lipases; dark blue circles, CC-NBS-LRR genes; black circles, downstream from the CC-NBS-LRR cluster, terpenoid cyclase genes. Grey lines represent one-to-one syntenic relations and serve only to better visualize the overall syntenic structure in the region. Gene ID numbers in red denote PAV genes. The cucumber and watermelon genome sequence and annotation was obtained from Phytozome [48] (data as of October 23rd 2012). Figure drawn to scale.

As mentioned above, the greatest density of resistance genes in the melon genome was found in a 760 kb fragment on linkage group V. If a cluster of lipoxygenase genes and one phytochelatin gene in the immediate vicinity of this fragment are also considered, the resulting 1.1 Mb region contains 97 annotated genes (MELO3C04235-4331) of which 38 (40%) are most probably involved in different aspects of biotic or abiotic stress responses (Figure 4). As well as 21 NBS-LRR-TIR and seven CC-NBS-LRR genes having homology with resistance genes, there are nine lipoxygenase genes, which may have roles in pest resistance and response to wounding, and one phytochelatin gene, that might be involved in resistance to metal poisoning [29,30]. These resistance genes are grouped in several clusters and 47% of them show PAV, not only in the analyzed melon lines but also when compared with the syntenic regions in two other cucurbits with sequenced genomes: cucumber, where only eight TIR-NBS-LRR, one CC-NBS-LRR, and no phytochelatins are found, and watermelon, with only five TIR-NBS-LRR, one CC-NBS-LRR, and no phytochelatins (Figures 3 and 5).

The highest concentration of PAV events detected in the melon genome was found in this region, with nearly 30% of all the genes partially or totally absent from at least one of the analyzed melon cultivars. In the vicinity of these genes, besides 18 PAV in stress response genes, there are also PAV genes in a cluster of GDSL lipases and a cluster of terpene cyclases, and eight additional PAV genes of unknown function. This region contains the longest continuous gene-containing PAV sequence found in melon: a fragment of 150 kb containing the genes MELO3C04318-4324 and completely absent from the C-836 cultivar, while a 90 kb fragment containing the genes 4319–4322 was absent from PI 161375, the *agrestis* parental of DHL92, and another fragment of 86 kb containing five resistance genes was absent from C-1012 (Figure 5).

Annotation of the reference melon genome has shown that the number of putative disease resistance genes of the NBS-LRR family in melon and cucumber is lower than in other plant species [8]. Although the expansion of the lipoxygenase gene family has been suggested as a mechanism to compensate for this reduction in cucumber [31], no such expansion has been observed in melon. However, our results show that a high degree of PAV polymorphism affects several families of disease resistance genes. It must also be taken into account that only absent genes relative to the DHL92 reference genome could be detected using our approach, so additional PAV events may be found if the resequencing reads are mapped against the genome of other cultivars. The 'pan-genome' concept has already been proposed to reflect the fact that single genome sequences do not seem to account for the full set of genes in plant species [32]. It is then possible that the full set of active melon R-genes cannot be obtained by analyzing the annotated genes from just one cultivar. In fact, different combinations of present and absent R-genes in a particular cultivar (or even in an individual plant) might well confer various levels of resistance to different pathogens or strains.

As an example, the *Vat* gen, an economically important gene conferring resistance to aphid colonization, has been cloned in PI 161375 and shown to be a CC-NBS-LRR gene syntenic to those found on the cluster of seven CC-NBS-LRR genes in the 1.1 Mb region of DHL92 [33,34]. Two alleles were found at this locus with different resistance specificities. However, it has been reported that the cloning of candidates in other melon accessions is particularly difficult, probably due to the existence of several sources of variability, including the variable complexity of the cluster of resistance genes depending on the genotype [35]. Related results have been found in other plant species. For example, nematode resistance mediated by the soybean *Rhg1* QTL has been found to be conferred by copy number variation: resistant plants contain ten tandem copies of a 31 kb segment that spans three dissimilar genes, all of which contribute to the resistance [7].

## Conclusions

This article presents the quantification of PAVs in the genome of melon by comparing the published reference genome to four additional melon cultivars. We found that multi-copy melon gene families are particularly affected by PAV polymorphism. This is in agreement with results in other species and it may respond to the need for multiple copies of a gene for new functions required for the evolution of the species. On further analyses, gene clusters appeared as hotspots of PAVs in the melon genome, with significant clustering in cases of biotic stress response-related genes. In particular, melon has two large clusters of sequences coding for NBS-LRR proteins where important resistance *loci* such as *Fom*1 or *Vat* map. The analysis of a 1.1 Mb region containing the *Vat* locus revealed the highest density of PAV events as well as the longest continuous gene-containing PAV sequence in melon.

Cucurbit genomes have the peculiarity that some gene families related to pathogen resistance are smaller than in most other plant species. Disease resistance genes are essential for the survival of the species and, therefore, they have also been the object of high selective pressure during domestication and breeding. This work reports the finding of high sequence variability involving genome fragments containing pathogen resistance genes in the melon genome. The variability of these clusters may well be the reason why, in some cases, it has been difficult to identify specific resistance genes in these regions. The results also show the importance of sequencing different cultivars of a particular cultivated plant species to characterize its genome variability, the understanding of its evolutionary history, and to help define breeding strategies.

## Methods

### Datasets

The sources of the *C. melo DH*L92 genome, its gene and functional annotations, and the Illumina resequencing read sets of DHL92 and its parental lines (T-111 and PI 161375) can be found in [8].

### Plant material and nucleic acid extraction

Seeds from the melon varieties C-836 and C-1012 were kindly provided by the Institute of Subtropical and Mediterranean Horticulture "La Mayora" (CSIC-UMA). Plants were grown in the greenhouse (diurnal temperature 28°C ± 2°C, nocturnal temperature 22°C ± 2°C, relative humidity 60°C ± 5°C) and young tender leaves were collected from five-week old plants. DNA was extracted from individual plants [36] and two DNA samples, one from each melon variety, were chosen for sequencing.

### DNA sequencing

From the two DNA samples, one from each melon variety, 2 μg from each sample were used to produce two paired-end libraries of average insert size 509 ± 20 bp, at the Centre Nacional d'Anàlisi Genomica (CNAG), Barcelona, Spain. These were sequenced using the Illumina GAIIx platform to produce two sets of paired-end reads of 152 bp. SCS software was used to create *bcl* files from the sequencer images and *qseq* files were then obtained using the Illumina OLB software with standard options. Finally, files in standard *fastq* format with Sanger (Phred +33) quality scores were produced. Only reads that passed the standard Illumina quality filtering were included in these files.

The fastq files containing the raw data were filtered using the DynamicTrim software (v.1.12) from the SolexaQA 2 package [37] so that each read was trimmed to the longest contiguous segment for which quality scores were greater than 15. The cutadapt_v1.0 software [38], with flag -O 6, was then used to remove Illumina adapter sequences and, to remove all reads shorter than 40 bp, the resulting files were processed using PRINSEQ lite software (v0.19.2). Non-paired single reads were removed using a perl script and finally the filterPCRdupl_v1.01 software [39] was used to remove any redundant copies from the PCR steps during library construction.

The Illumina paired-end reads from DHL92 and its parental lines, already available [8], were subjected to the same filtering procedures. In these three cases, the reads had been obtained by sequencing genomic DNA from several individuals.

*Fasta* files were obtained using the fastq_to_fasta software from the *FASTX-TOOLKIT* package [40], and the total length and N75 values were calculated using the count_fasta perl script found in [41].

### Paired-end mapping

Sequence reads, including those of DHL92 and its parental lines, were aligned to the melon reference genome sequence using the Burrows-Wheeler aligner (v0.6.1) [42]. First, the reference sequence was indexed using 'bwa index' with the '-a bwtsw' flag and the two fastq files for each melon variety aligned to the reference genome using 'bwa aln' with the default parameters. The *sam* files were then generated using 'bwa sampe' with the default parameters. The corresponding *bam* files were produced using the samtools software, *samtools view -bS mapping_file.sam -T ref_genome.fa -o mapping_file.bam*, and the resulting files sorted and indexed using *samtools sort* and *samtools index*.

### Identification and functional characterization of PAV genes

For each resequenced melon variety (including DHL92 and its parental lines), *samtools* software was used to generate a text file containing the number of Illumina reads that mapped at each gene location on the reference genome. Firstly, a text file was generated containing the coordinates of all DHL92 annotated genes as 'contig_or_scaffold_name:star_coord-end_coord gene_name'. A bash script was then used to feed the five sorted.bam files (one for each melon cultivar analyzed) to *samtools view* so that the following command was applied to every single contig and scaffold of the reference assembly: *samtools view -c -F 4 -q 1 mapping_file.sorted.bam contig_or_scaffold_name:start_coord-end_coord*.

The numbers of reads that mapped at each gene location for every melon variety were normalized by the total number of reads mapping the whole reference genome for each five melon varieties. These figures were calculated as follows: *samtools view -c -F 4 -q 1 mapping_file.sorted.bam*.

As a first approach to identify a putative PAV gene, a list was generated containing all genes for which at least one ratio between the mapping reads from two melon varieties was lower than 0.5 or higher than two. The resulting 3,034 genes were visually inspected using the IGV v2.1 software [43] to select those for which a probable deletion occurs in a melon variety. A total of 1,664 genes were selected in this way, but 492 had less than ten reads mapping in all five melon varieties and were discarded. As a second approach to identify putative PAV genes, all genes with less than six mapped reads from at least one variety and more than 29 mapped reads from at least another variety were selected. This gave 594 genes, of which 558 were already present in the list of candidate PAV genes obtained using the first approach. After visual inspection using IGV, the 36 additional genes were added to that list. Six additional putative PAV genes, independently detected while IGV-browsing through the mapping files, were included in the final list that contained 1,214 candidate PAV genes.

The functional characterization of the selected genes was using the annotation of the melon assembly than can be found on the MELONOMICS webpage [18]. Additionally, a list of the 1,214 predicted protein sequences coded by the putative PAV genes was analyzed using the Blast2GO software [44] using the following consecutive steps: a) blastp with e-value 1e-10, database nr, and HSP length cut-off 33; b) annotation step with e-value 1–10, cut-off 55, Go weight 5, and hsp 0; c) ANNEX; d) Interproscan; e) Merge Interproscan to annotation; f) get KEGG maps; g) GO-slim; and h) combined graph. Finally, information from the melon phylome [19] was also used to tentatively assign putative functions to ten genes, based on phylogenetic relationships.

### Characterization of multi-copy gene families and gene clusters

A local blast database of all *C. melo* genes was built and every gene subjected to a BLASTN search to identify homologous genes using a 1e-20 e-value threshold. All resulting pairs of homologous gene fragments showed at least 69% nucleotide identity. To refine this search to include only genes for which a significant fraction of the sequence shows homology to another gene, a filter was applied to keep only blast results for which the query and subject coverages were greater than 40%. The genes absent from the final filtered list were considered as singletons, while all other genes were grouped in families based on the BLASTN results.

A gene cluster was defined as a set of two or more homologous genes close to each other, with less than nine genes allowed between homologs for these to be considered as part of the same cluster. In a few cases, the nine gene criterion was relaxed to allow the inclusion of a gene in a cluster, based on visual inspection of the annotated region.

### Validation of PAV

A minimal tiling path of BAC clones spanning 713 kb of CM3.5_scaffold00003, and comprising the melon genes MELO3C004287-MELO3C004347, was obtained using available BAC-end sequence data [45], a previously built BAC-based physical map [46], and the melon genome reference sequence. Based on this information, nine BACs from a *Bam*HI genomic library [24] and one additional BAC from a random-shear library [44] were chosen.

Pulsed-field gel electrophoresis was used to estimate the insert lengths (PFGE). BAC DNA was extracted from the selected clones as described in [47], and the genomic inserts released by *Not*I enzymatic digestion (Roche Applied Science). These were separated on 1% agarose by PFGE using 0.5 × TBE at 12.5°C, and a pump setting of 80, 120 degree angle, 6 V/Cm, 5 s initial, and a 15 s final pulse time for 16 h. The insert lengths were quantified using the PGF lambda ladder marker (New England Biolabs). The average lengths and standard deviations were calculated using values obtained from 3–7 electrophoretic runs for each clone.

Oligonucleotides designed to flank the candidate regions were used to confirm the putative PAVs by PCR, using genomic DNA from the studied varieties as well as from the reference genome, either genomic DNA or DNA from BAC clones. DHL92, T-111, PI 161375, C-836 and C-1012 DNA was obtained from tender young leaves using the Nucleo-Spin Plant II (Macherey-Nagel). DNA from BAC clones was obtained as described above. RANGER DNA Taq polymerase (Bioline) was used for PCRs. The amplified bands were purified and sequenced.

### Identification of *Cucumis sativus* and *Citrullus lanatus* syntenic regions

Files with the predicted proteins of the cucumber and watermelon genome assemblies and their .gff3 annotation files were downloaded from Phytozome [48] (data as of October 23rd 2012). Then, BLASTP was used with e-value 1e-10 and 1) database: melon proteins, query: cucumber proteins 2) database: melon, query: watermelon proteins 3) database: melon proteins, query: melon proteins, 4) database: cucumber proteins, query: cucumber proteins, and 5) database: watermelon proteins, query: watermelon proteins. The results from these analyses were combined to produce a melon-cucumber and a melon-watermelon blast files. The melon, cucumber, and watermelon .gff3 files were modified so that they contained only gene annotations in the following format: 'sp# gene_name starting_position ending_position', where 'sp#' is 'csN', 'clN', or 'cmN', and N is the number of the corresponding scaffold/chromosome. These modified .gff3 files were combined to produce a melon-cucumber and a melon-watermelon gff3 files. The combined blast and gff3 files were processed by MCScanX software [49], scanning the melon, cucumber, and watermelon genomes to identify putative homologous regions and to align those regions using genes as anchors.

## Availability of supporting data

The data sets supporting the results of this article (*C. melo* C-836 and C-1012 Illumina paired-end sequences) are available in the EBI SRA repository, accession number ERP002386, http://www.ebi.ac.uk/ena/data/view/ERP002386.

## Competing interests

The authors declare that they have no competing interests.

## Authors’ contributions

VMG performed all non-experimental analyses and drafted the manuscript, NA conducted all experimental work (genomic and BAC DNA extraction, genotyping, PFGE, and validation of PAVs), EC helped to process the Illumina data and to obtain the mapping files, offered overall bioinformatics support, and participated in the discussion of the results, PP conceived and coordinated the project, participated in the discussion of the results and drafted the manuscript. All authors read and approved the final manuscript.

## Supplementary Material

Additional file 1 Genotyping of the resequenced varieties. Click here for file

Additional file 2: Table S1 List of putative PAV genes with functional annotations. Click here for file

Additional file 3: Table S2 Distribution of PAV genes in functional categories. Click here for file

Additional file 4: Figure S1 Tiling path of BAC clones spanning 713 kb of CM3.5_scaffold00003, and comprising the melon genes MELO3C004287-MELO3C004347. Click here for file

Additional file 5: Figure S2 Estimated lengths of BACs from a tiling path comprising the melon genes MELO3C004287-MELO3C004347. Click here for file
