# Supplementary material for: High presence/absence gene variability in defense-related gene clusters of Cucumis melo
Source: BMC Genomics. 2013 Nov 12;14:782. doi: 10.1186/1471-2164-14-782 (PMC3845527; doi:10.1186/1471-2164-14-782)
Supplement: Additional file 1 — Genotyping of the resequenced varieties. [file 1471-2164-14-782-S1.pdf]

Genotyping of the resequenced varieties

Little information regarding the genotype of C-836 and C-1012 is available other than their tentative adscription, based on their phenotypic traits to, respectively, the *agrestis* and *melo* subspecies. Therefore, a phylogenetic tree was built to position those cultivars relative to five other melon varieties: both DHL92 parentals, two subsp. *melo* cultivars (PI 124112 [1], and Védrantais) and one *agrestis* cultivar (*C. trigonus* (Ames 24297) [1]). Eighteen SSR markers previously used to study genetic diversity in a collection of melon cultivars were used [2]. These markers had been developed from cucumber and melon sequence data and were chosen so that at least one SSR locus was represented in each of the 12 linkage groups defined in [3]. The molecular sizes of the amplified bands used to build the tree can be found below. The resulting tree confirms that C-836 and C-1012 belong, respectively, to the *agrestis* and *melo* subespecies.

Methods

Genotyping of the C-836 and C-1012 melon varieties was with 18 SSR markers previously used to study genetic diversity in a collection of melon cultivars [2].

Genomic DNA was used for PCR amplification of the genetic markers. Genomic DNA from five other melon varieties was included to build a phylogenetic tree of the *C. melo* species in which to position our sequenced melon varieties. The additional samples of melon genomic DNA were kindly provided by the Plant Genetics Department of CRAG and had been obtained from the following varieties: T-111 (Piel de Sapo), PI 161375, PI 124112 [1], Ames 24297 (*C. trigonus*) [1], and Védrantais. Each DNA sample was obtained from a single plant.

All PCR reactions were in a total volume of 15 µl with 5-10 ng of genomic DNA and 2mM MgCl2, and the forward primer labeled with IDR-800. The cycling conditions were: a) 94°C, 1 min; b) 35 cycles of 94°C, 30 s, 48-51°C, 30 s, and 72°C, 1 min; c) 72°C, 5 min.

After the addition of 5 µl of loading buffer to the PCR mix followed by denaturation at 95 °C for 3 min, 0.8 µl from each sample were loaded into a LICOR 4300 DNA Analyzer for electrophoresis (1500V, 35mA and 31W at 50ºC) until the PCR products were visible. The molecular weight of the amplicons was estimated using the IRD-800 50-350 sizing standard.

The phylogenetic tree of the melon varieties was built using the PowerMarker and Mega5 software solutions, applying Nei's standard genetic distance definition and the Neighbor-Joining method for tree building.

Bibliography

1. Monforte AJ, Iban E, Abad S, Arús P: **Inheritance mode of fruit traits in melon: Heterosis for fruit shape and its correlation with genetic distance**. *Euphytica* 2005, 144: 31-38.

2. Monforte AJ, Garcia-Mas J, Arús P: **Genetic variability in melon based on microsatellite variation**. *Plant Breeding* 2003, 2: 153-157.

3. Oliver M, Garcia-Mas J, Cardús M, Pueyo N, López-Sesé AI, Arroyo M, Gómez-Paniagua H, Arús P, De Vicente MC: **Construction of a reference linkage map for melon**. *Genome* 2001, 44: 836-845.

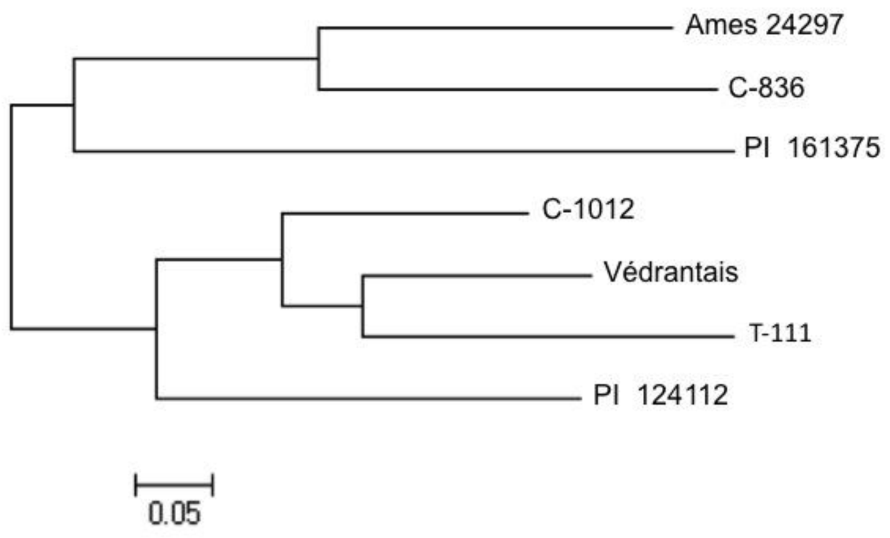

Figure 1. Phylogeny tree of seven melon cultivars, including C-836, C-1012 and both DHL92's parentals. Tree built from SSR genotyping using PowerMarker and Mega5, according to Nei's standard genetic distance definition and the Neighbor-Joining method for tree building.

Table 1. Experimental data used to build the phylogenetic three of seven melon cultivars.

sample	CMAG59	CSGA57	CMGA128	CSAT425b	CMGA15	CMAT35	CMTAA166	CMGA104	CMTC160a+b	CMCCA145
**T111**	126/126	204/204	116/116	106/106	146/146	110/110	183/183	133/133	205/205	140/140
**PI**	132/132	210/210	118/118	132/132	144/144	116/116	162/162	123/123	218/218	147/147
**TRI**	132/132	206/206	118/118	104/104	144/144	114/114	155/155	145/145	213/213	147/147
**CAL**	124/124	206/206	122/122	106/106	146/146	110/110	162/183	140/140	210/210	147/147
**VED**	124/124	210/210	116/116	106/106	146/146	110/110	162/162	127/127	205/205	140/140
**CV**	134/134	206/206	000/000	110/115	148/148	114/114	145/145	129/129	213/213	147/147
**IRK**	124/132	204/210	116/116	104/104	146/146	110/110	162/162	138/140	205/218	140/140

sample	CMTC47	NR22	CMGT108	CMTA134a	CSCCT571	CMTC168	CSAT425a	CMTC123
**T111**	166/166	157/157	186/186	140/140	215/215	217/217	102/102	100/100
**PI**	164/164	162/162	182/182	150/150	207/207	208/208	090/090	098/098
**TRI**	210/210	157/157	182/182	135/135	205/205	205/205	095/095	100/100
**CAL**	158/158	156/160	184/184	138/138	215/215	217/217	092/092	100/100
**VED**	166/166	157/157	182/182	155/155	215/215	217/217	090/090	100/100
**CV**	158/188	157/164	184/184	135/135	205/205	205/205	092/092	100/100
**IRK**	168/170	157/159	184/186	148/150	215/215	217/217	090/092	100/100
