# Supplementary material for: High presence/absence gene variability in defense-related gene clusters of Cucumis melo
Source: BMC Genomics. 2013 Nov 12;14:782. doi: 10.1186/1471-2164-14-782 (PMC3845527; doi:10.1186/1471-2164-14-782)
Supplement: Additional file 4: Figure S1 — Tiling path of BAC clones spanning 713 kb of CM3.5_scaffold00003, and comprising the melon genes MELO3C004287-MELO3C004347. [file 1471-2164-14-782-S4.pdf]

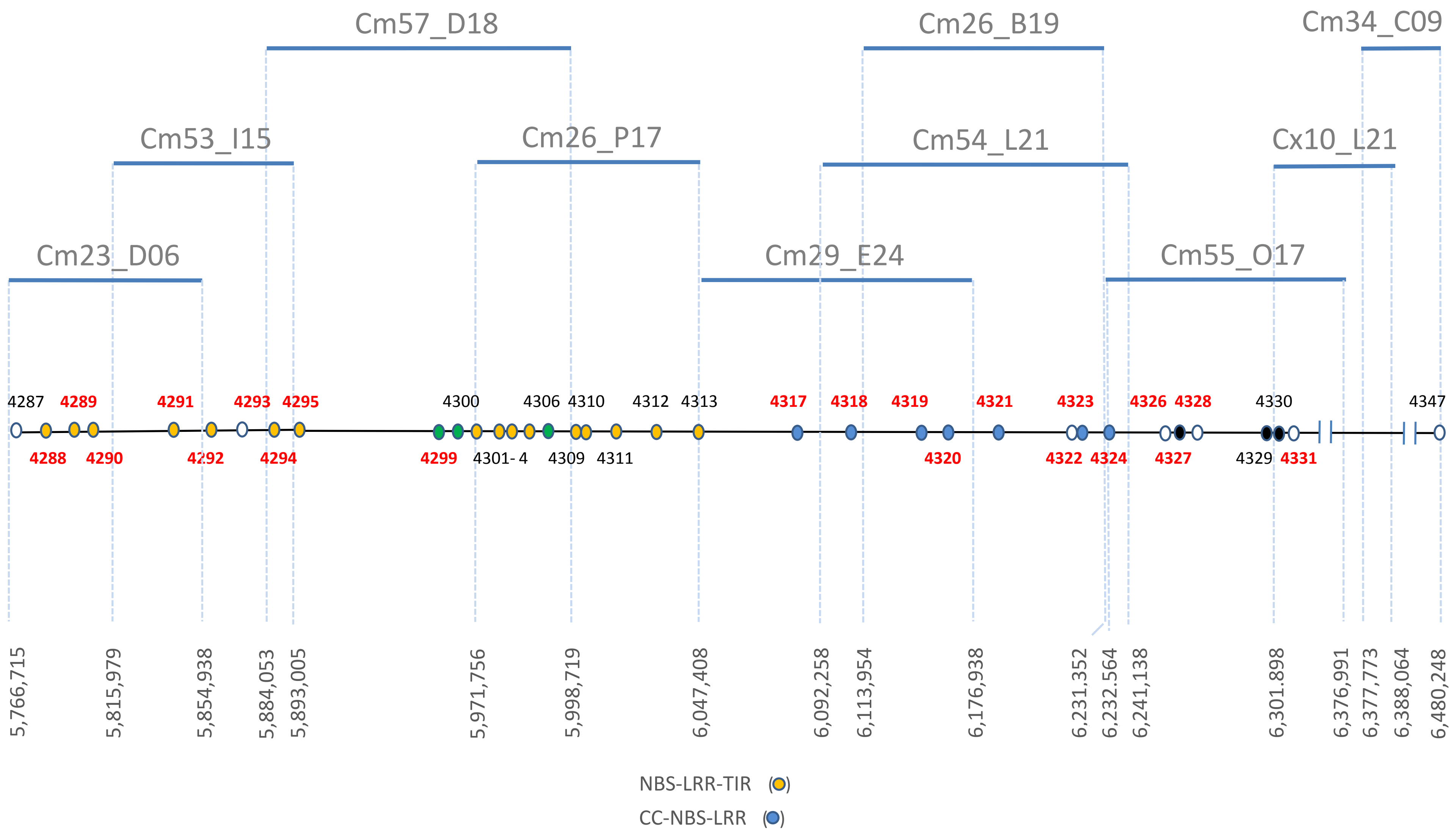

Figure S1

Tiling path of BAC clones spanning 713 kb of CM3.5_scaffold00003, and comprising the melon genes MELO3C004287-MELO3C004347. Genes are represented by colored circles. The same color is used for all genes sharing the same biological function. Gene IDs in red represent PAV genes. The source of BAC clones and BAC-end sequences can be found in [45]. Figure drawn to scale.
