# Supplementary material for: High presence/absence gene variability in defense-related gene clusters of Cucumis melo
Source: BMC Genomics. 2013 Nov 12;14:782. doi: 10.1186/1471-2164-14-782 (PMC3845527; doi:10.1186/1471-2164-14-782)
Supplement: Additional file 5: Figure S2 — Estimated lengths of BACs from a tiling path comprising the melon genes MELO3C004287-MELO3C004347. [file 1471-2164-14-782-S5.pdf]

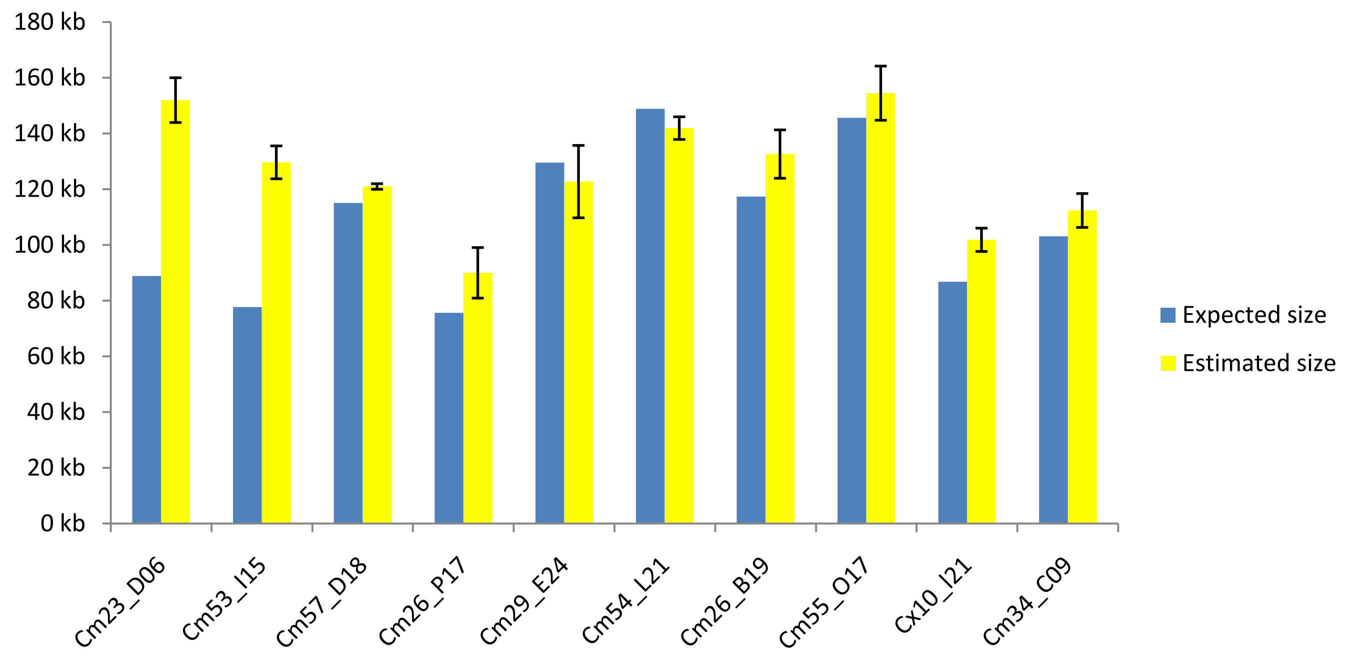

Figure S2

Estimated lengths of BACs from a tiling path comprising the melon genes MELO3C004287 - MELO3C004347. Expected sizes were calculated by mapping the BAC-end sequences to the melon reference genome assembly. 'Estimated sizes' refers to the actual BAC lengths estimated averaging the result of 3-7 pulse-field electrophoretic runs for each clone.
